# Pharmacological and Non-Pharmacological Interventions for Non-Suicidal Self-Injury: A Narrative Review

**DOI:** 10.31083/AP48183

**Published:** 2026-04-08

**Authors:** Jieying Tan, Lulu Zhang

**Affiliations:** ^1^The First School of Clinical Medicine, Guangdong Medical University, 524023 Zhanjiang, Guangdong, China; ^2^Department of Psychiatry, Guangzhou First People’s Hospital, Guangdong Medical University (Guangzhou First People’s Hospital), 510180 Guangzhou, Guangdong, China

**Keywords:** non-suicidal self-injury, psychotherapy, drug therapy, electric stimulation therapy, digital health, adolescents

## Abstract

Non-suicidal self-injury (NSSI), particularly among youth, is an increasing, global, public health concern. Some studies have shown that NSSI is an independent risk factor for suicidal behavior. However, evidence-based treatment guidelines remain underdeveloped. We searched PubMed, Web of Science, and the China National Knowledge Infrastructure (CNKI) for studies on adolescent or adult NSSI published between January 2005 and August 2025 to try to determine the most effective treatment strategies. We included 50 studies. Interventions were categorized into psychotherapy, pharmacotherapy, neuromodulation, and digital health approaches. Psychotherapy, especially dialectical behavior therapy (DBT), showed the most consistent efficacy in reducing NSSI frequency and improving emotion regulation. Adjunctive pharmacotherapy with antidepressants and atypical antipsychotics alleviated comorbid symptoms, but caution is warranted in younger patients due to potential risks. Neuromodulation techniques, including repetitive transcranial magnetic stimulation (rTMS) and transcranial electrical stimulation (TES), may reduce NSSI through modulation of prefrontal cortex activity, and deep brain stimulation (DBS) show promise for treatment-resistant cases. Digital tools such as mobile applications, ecological momentary assessment, and artificial intelligence (AI)-assisted technologies enable real-time monitoring and risk prediction but remain complementary to traditional crisis intervention. Ultimately, in this review, we highlight the importance of multimodal and individualized strategies in managing NSSI and provide insights to guide future clinical practice and research.

## Main Points

(1) Dialectical behavior therapy (DBT) shows the most consistent efficacy among psychotherapies for reducing non-suicidal self-injury (NSSI).

(2) Combined psychotherapy and pharmacotherapy enhances NSSI symptom improvement.

(3) Repetitive transcranial magnetic stimulation (rTMS) and transcranial direct current stimulation (tDCS) show emerging potential as neuromodulation treatments for NSSI.

(4) Digital tools enable real-time monitoring and early prediction of self-injury risk.

## 1. Introduction

Non-suicidal self-injury (NSSI) is defined as the deliberate, self-inflicted damage to body tissue without suicidal intent and outside socially accepted norms [1]. It encompasses behaviors such as cutting, scratching, hitting, biting, and interfering with wound-healing [2], with noted gender differences; females more often engage in scratching and pinching, whereas males tend toward hitting [2]. Beyond immediate physical damage, NSSI frequently leads to permanent scarring, which is associated with heightened negative self-cognitions [3, 4]. Affected individuals also experience stronger implicit and explicit negative biases, and stigma-related shame compounds long-term psychological distress [5]. Moreover, NSSI is linked to elevated rates of comorbid psychiatric disorders, including depression, anxiety, bipolar disorder, substance use, attention deficit hyperactivity disorder (ADHD), and post-traumatic stress disorder (PTSD) [6]. Notably, nearly 60% of individuals with a lifetime history of NSSI meet criteria for at least one psychiatric disorder [6], underscoring its substantial disease burden.

The prevalence of NSSI is particularly high among youth. In clinical adolescent samples, past-year engagement in NSSI reaches 60%, with almost half reporting repetitive behavior [7]. A meta-analysis reported that the overall prevalence of NSSI among non-clinical adolescents worldwide was 17.2% [8], with Chinese preadolescent rates of around 13.6% [9]. The incidence of NSSI peaks between ages 15 and 17 and gradually declines thereafter [10], though adult psychiatric outpatient rates remain notable (e.g., 8.1% in a Norwegian sample) [11]. Adolescence, a critical period of biopsychosocial development, is marked by emotional dysregulation that can precipitate NSSI and increase suicide risk [12, 13]. Indeed, NSSI is a documented predictor of suicidal ideation and attempts [14, 15, 16], with each additional act of self-injury raising subsequent suicide attempt risk sevenfold [17]. In one study, over 30% of adolescents with NSSI also exhibited suicidal behaviors [18], which suggests the urgent need for effective intervention.

Research on NSSI mechanisms has implicated genetic, emotional, cognitive, and neurobiological factors, including deficits in emotion regulation, altered pain processing, and impulsive aggression [19, 20]. Despite theoretical advances, evidence-based treatment guidelines have remained underdeveloped, and current management often draws from approaches used for other mental disorders. Available interventions include: (a) psychotherapy, such as dialectical behavior therapy (DBT), cognitive behavioral therapy (CBT), mentalization-based treatment (MBT), and acceptance and commitment therapy (ACT), which currently has the strongest empirical support and is considered the core psychosocial component of care [21, 22, 23, 24, 25]; (b) adjunctive pharmacotherapy (e.g., selective serotonin reuptake inhibitors and second-generation antipsychotics) may address comorbid symptoms but carries concerns about side effects and limited efficacy for NSSI, per se [26, 27, 28]; (c) neuromodulation techniques (e.g., transcranial magnetic stimulation [TMS], transcranial electrical stimulation [TES], and deep brain stimulation [DBS]) represent emerging options [20]; and (d) digital health technologies offer a convenient way to support the delivery of healthcare services [29], though modalities like magnetic seizure therapy and artificial intelligence (AI)-assisted tools require further validation.

This narrative review synthesized current evidence on NSSI interventions across psychological, pharmacological, neuromodulation, and digital domains, with the aim of determining the most effective treatment strategies and guiding clinical practice and future research.

## 2. Methods

Given the methodological heterogeneity across studies on treatments for NSSI, this narrative review synthesized existing evidence and provided a comprehensive overview of advances in this field, rather than producing a systematic analysis. We conducted an exploratory search of PubMed (https://pubmed.ncbi.nlm.nih.gov/), Web of Science (https://www.webofscience.com/), and the China National Knowledge Infrastructure (CNKI) database (https://www.cnki.net/), covering publications from January 2005 to August 2025.

In PubMed and Web of Science, a blend of the following search terms was used: non-suicidal self-injury; NSSI; intervention; treatment; management; psychotherapy; pharmacotherapy; and neuromodulation. In CNKI, the search was conducted using the Chinese equivalent search terms that included non-suicidal self-injury, treatment, and intervention. All key terms were combined in flexible configurations using Boolean logic, such that one term related to NSSI and one term related to intervention were searched, and subsequently, narratively reviewed.

Studies were included if they: (1) included original research articles; (2) included systematic reviews; (3) included clinical trials; (4) reported NSSI as a primary or secondary outcome; (5) did not report NSSI-specific data, but the primary endpoint was broader self-harm or suicidal behavior. Studies were excluded if they: (1) included animal research; (2) were published in languages other than English or Chinese.

The present narrative review included 50 studies: 19 on psychotherapeutic interventions, 15 on pharmacological interventions, 10 on neuromodulation techniques, and 6 on digital health technologies.

Because of the heterogeneity of study designs, we assessed methodological quality using the Quality Assessment with Diverse Studies (QuADS) tool. This tool has demonstrated strong reliability and usability for reviews involving mixed or multi-method evidence [30]. For each study, the final score was calculated and expressed as a percentage of the maximum possible score using the following formula: final score = total score of each study/total criteria score ×100% [30, 31]. In addition, the strength of evidence for all included studies was evaluated using the Oxford Centre for Evidence-Based Medicine (OCEBM) Levels of Evidence [32]. Quality assessments and levels of evidence are presented in Table 1 (Ref. [21, 24, 33, 34, 35, 36, 37, 38, 39, 40, 41, 42, 43, 44, 45, 46, 47, 48, 49]), Table 2 (Ref. [27, 50, 51, 52, 53, 54, 55, 56, 57, 58, 59, 60, 61, 62, 63]), Table 3 (Ref. [64, 65, 66, 67, 68, 69, 70, 71, 72, 73]), and Table 4 (Ref. [29, 74, 75, 76, 77, 78]).

**Table 1.** **A summary of psychotherapy studies in this narrative synthesis**.

Author	Language	Study design	Study subject	Sample	Intervention	Intervention (+ follow-up)	Primary outcomes	Main findings	Percentage total score	Level
Asarnow *et al*., 2021 [33]	English	RCT	Adolescents (aged 12–18)	IG: 86 CG: 87	IG: DBT CG: IGST	6 months + 6 months follow-up	Remission rate of NSSI; Emotion regulation	DBT led to significantly greater improvement in youth emotion regulation than IGST;	94.87%	Level 2
								DBT was superior to IGST in achieving self-harm remission at both post-treatment (44.2% vs. 27.3%) and follow-up (49.3% vs. 29.7%)		
Berk *et al*., 2024 [34]	English	RCT	Adolescents	IG: 86 CG: 87	IG: DBT CG: IGST	6 months + 6 months follow-up	Remission rate of suicide attempts; NSSI remission	DBT significantly increased remission of suicide attempts and reduced relapse;	94.87%	Level 2
								DBT showed higher recovery rates at 3–12 months		
McMain *et al*., 2022 [35]	English	RCT	Adults (aged 18–60) diagnosed with BPD	DBT-6 months: 120 DBT-12 months: 120	DBT	DBT-6: 6 months + 18 months follow-up	Frequency of NSSI	Both DBT durations reduced NSSI;	94.87%	Level 2
						DBT-12: 12 months + 12 months follow-up		6-month DBT was non-inferior to 12-month;		
								DBT-6 showed more rapid reductions in BPD symptoms and general psychopathology at 6 months		
Santamarina-Perez *et al*., 2020 [36]	English	RCT	Adolescents (aged 12–17)	IG: 18 CG: 17	IG: DBT-A CG: TAU + GS	16 weeks	Frequency of NSSI	DBT-A more effectively reduced NSSI and improved functioning compared with TAU + GS	79.49%	Level 2
Mehlum *et al*., 2019 [37]	English	RCT	Adolescents with at least two borderline personality disorder traits	IG: 37 CG: 34	IG: DBT-A CG: EUC	19 weeks + 3 years follow-up	Frequency of NSSI	At the 3 years follow-up, the DBT-A group maintained a significantly lower frequency of self-harm episodes compared to the EUC group;	92.30%	Level 2
								Receiving more than 3 months of additional treatment after the trial phase was associated with further enhanced outcomes (84% fewer NSSI episodes) specifically in the DBT-A group		
Simon *et al*., 2022 [38]	English	RCT	Adults (≥18 years)	IG1: 6230 IG2: 6227 CG: 6187	IG1: Care management IG2: Skills training (DBT) CG: Usual care	12 months + 18 months follow-up	Time to first fatal or nonfatal self-harm within 18 months	Care management group showed no significant difference in the risk of self-harm compared with usual care group (3.27% vs. 3.10%);	94.87%	Level 2
								Skills training group had a significantly increased risk of self-harm compared with usual care group (3.92% vs. 3.10%)		
Sinyor *et al*., 2020 [39]	English	RCT	Youth (aged 16–26)	IG: 12 CG: 12	IG: BCBT CG: Minimally-directive supportive psychotherapy	10 sessions over 15 weeks (acute phase) + 3 booster sessions at 6, 9, 12 months	Frequency of repeat NSSI	BCBT group had significantly fewer instances of repeat self-harm during acute treatment (11% vs. 30%)	82.05%	Level 3
Lou *et al*., 2024 [40]	Chinese	RCT	Adolescents (aged 11–18)	IG: 46 CG: 46	IG: ICBT CG: Traditional nursing intervention	5 weeks	Positive and negative emotion; Depression and anxiety severity; NSSI behavior	ICBT group showed significantly higher positive affect and lower negative affect, depression, anxiety, and NSSI behavior scores	74.36%	Level 2
Chen *et al*., 2025 [21]	English	Systematic review and network meta-analysis	Children and adolescents (aged ≤25)	6496 participants	Psychotherapies (e.g., DBT, CBT); Pharmacotherapies (e.g., SSRIs, SNRIs); Combination therapies; Control conditions (placebo, TAU, no treatment)	Treatment duration varied across trials	Frequency of NSSI	DBT was most efficacious in reducing NSSI frequency, superior to TAU;	87.20%	Level 1
								CBT was less efficacious than other therapies and showed a worse tendency for NSSI;		
								SSRIs increased NSSI frequency significantly within the first 3 months but reduced it after 3 months;		
								Interventions aggravated NSSI in patients with depression but reduced it in those with self-harm		
Kaess *et al*., 2020 [41]	English	RCT	Adolescents (aged 12–17)	IG: 37 CG: 37	IG: CDP CG: TAU	2–4 months + 10 months follow-up	Frequency of NSSI	Both groups showed 50% reduction in the frequency of NSSI within the past 6 months at 10 months follow-up and there was no significant difference;	97.43%	Level 2
								CDP group showed faster reduction in NSSI frequency within the past month at mid-treatment and achieved similar outcomes with significantly fewer therapy sessions than TAU		
Rockstroh *et al*., 2023 [42]	English	Long-term follow-up study of a previous RCT	Adolescents (aged 12–17)	IG:34 CG: 36	IG: CDP CG: TAU	2–4 years follow-up	Frequency of NSSI	Both groups showed further significant reductions in NSSI frequency (84% reduction) over the long term	94.87%	Level 3
Griffiths *et al*., 2019 [43]	English	RCT	Adolescents (aged 12–18)	IG: 26 CG: 27	IG: MBT-Ai + TAU CG: TAU	12 weeks + 12, 24, 36 weeks follow-up	Self-reported self-harm; Self-harm-related emergency department presentations	Self-harm and self-harm-related emergency department presentations decreased over time in both groups and there was no statistically significant difference	82.05%	Level 2
Mohajerin *et al*., 2025 [44]	English	RCT	Adolescents (aged 11–17) diagnosed with BPD	IG1: 45 IG2: 46	IG1: MBT-A IG2: UP-A	MBT-A (12 months + 36 months) UP-A (3 months + 36 months)	Severity of borderline symptoms; Emotion regulation; Frequency of NSSI	Both groups significantly reduced borderline symptom severity and self-harm over time;	92.30%	Level 2
								UP-A was superior to MBT-A in reducing emotional dysregulation, despite being shorter and less intensive;		
								Treatment gains declined over the 36-month follow-up, with remission rates near zero at 36 months for both groups		
Beck *et al*., 2020 [45]	English	RCT	Adolescents (aged 14–17) with BPD or subthreshold BPD	IG: 56 CG: 56	IG: MBT-G CG: TAU	1 year	Severity of borderline symptoms; Self-harm behaviors	Remission rates of symptoms were identical (29%) in both groups;	92.30%	Level 2
								Both groups showed comparable yet limited effectiveness in reducing self-harm behavior		
Jørgensen *et al*., 2021 [46]	English	RCT	Adolescents (14–17) with BPD or subthreshold BPD	IG: 56 CG: 56	IG: MBT-G CG: TAU	1 year + 3, 12 months follow-up	Severity of borderline symptoms; Self-harm behaviors	During the follow-up period, both groups showed low symptom-remission rates, with no significant differences between them;	92.30%	Level 2
								During follow-up, neither treatment demonstrated strong effectiveness in reducing self-injury behaviors		
Mohajerin *et al*., 2024 [47]	English	RCT	Adults with comorbid BPD and ASPD	IG: 55 CG: 53	IG: MBT CG: UP	1 year + 36 months follow-up	Psychopathy traits: meanness, boldness, disinhibition; Impulsivity, anger expression and self-harm behaviors	Both treatments led to short-term reductions in psychopathy traits, BPD/ASPD symptoms, anger, impulsivity, and self-harm;	82.05%	Level 2
								UP showed more durable effects than MBT, particularly on emotion dysregulation;		
								Both groups showed nearly complete relapse to baseline symptom levels by the 36-month follow-up		
Yuan *et al*., 2024 [24]	English	Retrospective Controlled Study	Adolescent (aged 13–18)	IG: 36 CG: 36	IG: ACT + Routine psychological support CG: Routine psychological support	6 weeks + 12 weeks follow-up	Cognitive emotion regulation; NSSI behavior and function	At 6-week and 12-week, ACT group showed significantly higher scores in positive emotion regulation and cognitive fusion;	74.36%	Level 3
								ACT group showed significantly lower scores in negative emotion regulation, NSSI behavior and function		
Chen *et al*., 2024 [48]	Chinese	RCT	Adolescent (aged 10–19) with mood disorders	IG: 40 CG: 40	IG: ACT + Routine nursing CG: Routine nursing	3 weeks + 1 month follow-up	Negative emotions; Coping style; NSSI behaviors	IG groups showed significantly lower scores in anxiety, depression and negative coping;	74.36%	Level 2
								At 1-month follow-up, the IG had a significantly lower incidence of NSSI behavior (12.5% vs. 32.5%)		
Wang *et al*., 2023 [49]	Chinese	RCT	Adolescent (aged 12–18)	IG: 23 CG: 23	IG: ACT + Conventional drug therapy CG: Conventional drug therapy	8 weeks	Anxiety; Depression; Psychological flexibility; Incidence of NSSI	IG groups showed significantly lower scores in anxiety, depression, and measures of psychological inflexibility;	71.80%	Level 2
								IG group showed a significant reduction in the incidence of NSSI		

RCT, randomized controlled trial; IG, intervention group; CG, control group; DBT, dialectical behavior therapy; IGST, individual and group supportive therapy; BPD, borderline personality disorder; DBT-A, adapted dialectical behavior therapy; TAU, treatment as usual; GS, group sessions; EUC, enhanced usual care; BCBT, brief cognitive behavioral therapy; ICBT, intensive cognitive behavioral therapy; CDP, cutting-down program; MBT-A, mentalization-based treatment for adolescents; ASPD, antisocial personality disorder; UP-A, unified protocol for adolescents; SSRIs, selective serotonin reuptake inhibitors; SNRIs, serotonin and norepinephrine reuptake inhibitors; MBT-Ai, mentalization-based treatment for adolescent individual; MBT-G, mentalization-based treatment for group; UP, unified protocol; ACT, acceptance and commitment therapy; NSSI, non-suicidal self-injury.

**Table 2.** **A summary of pharmacotherapy studies in this narrative synthesis**.

Author	Language	Study design	Study subject	Sample	Intervention	Intervention (+ follow-up)	Primary outcomes	Main findings	Percentage total score	Level
Liu *et al*., 2025 [50]	English	RCT	Adolescents with depression	IG1: 50 IG2: 50	IG1: Sertraline + DBT IG2: Sertraline + CBT	12 weeks + 6 months follow-up	Frequency of NSSI	Sertraline + DBT group had significantly higher proportion of participants with no NSSI at 6 months (57.8% vs. 32.6%)	97.43%	Level 2
Liu *et al*., 2022 [51]	English	Meta-analysis	Adolescents with depression	1232 participants	IG: Fluoxetine + CBT CG: Fluoxetine	Treatment duration varied by included studies + follow-up to 1 year for recurrence rate	Response rate; Adverse reactions; Suicide/NSSI incidence; Recurrence rate; Depression scale scores	Fluoxetine + CBT showed significantly higher response rate, lower adverse reactions, lower suicide/NSSI incidence, lower 1 year recurrence rate, and greater reduction in depression scores compared to fluoxetine alone	94.87%	Level 1
Davey *et al*., 2019 [27]	English	RCT	Youth (aged 15–25) with moderate-to-severe MDD	IG: 76 CG: 77	IG: CBT + Fluoxetine (20–40 mg/day) CG: CBT + Placebo	12 weeks	MADRS; GAD-7; Suicidal ideation; Suicidal behavior; Suicide attempts; NSSI	Fluoxetine + CBT did not lead to greater reduction in core depressive symptoms;	97.43%	Level 2
								Fluoxetine + CBT showed a significantly greater reduction in anxiety symptoms than the placebo + CBT;		
								Fluoxetine + CBT had a higher incidence of NSSI compared to CBT + Placebo (29% vs. 15%), but not statistically significant		
Yan *et al*., 2025 [52]	Chinese	RCT	Adolescents with MDD	IG: 60 CG: 60	IG: Fluvoxamine + Simplified CBT CG: Fluvoxamine + Routine psychological intervention	8 weeks	Depression severity; Mental health; Frequency of NSSI	Fluvoxamine + simplified CBT showed significantly better outcomes in depression, psychological sub-health, NSSI severity, and resilience compared to the control group	82.05%	Level 2
Yuan *et al*., 2022 [53]	Chinese	RCT	Adolescents with depressive episodes	IG: 31 CG: 31	IG: Escitalopram + CBT CG: Escitalopram	8 weeks	Anxiety/depression; Frequency of NSSI	Escitalopram + CBT improved anxiety/depression and reduced NSSI	82.05%	Level 2
Qu *et al*., 2025 [54]	Chinese	RCT	Adolescents with depression	IG: 38 CG: 38	IG: Quetiapine (up to 200 mg/day) + Sertraline (100 mg/day) CG: Sertraline (100 mg/day)	4 weeks	Frequency of NSSI; Anxiety; Depression	Quetiapine + sertraline significantly reduced NSSI, anxiety and depression	74.36%	Level 2
Chen *et al*., 2024 [55]	Chinese	RCT	Adolescents with depression	IG: 30 CG: 30	IG: Low-dose olanzapine + Sertraline (25–50 mg/day) CG: Sertraline (25–50 mg/day)	4 weeks	Frequency of NSSI; Anxiety; Depression	Low-dose olanzapine + sertraline significantly reduced NSSI, anxiety and depression	79.49%	Level 2
Hu, 2025 [56]	Chinese	RCT	Adolescents with depression	IG: 33 CG: 33	IG: Low-dose olanzapine (2.5–5.0 mg/night) + Sertraline (50–150 mg/day) + DBT	6 weeks	NSSI; Anxiety; Depression	Low-dose olanzapine + Sertraline + DBT significantly reduced NSSI, anxiety and depression	71.80%	Level 2
					CG: Sertraline (50–150 mg/day) + DBT					
Nickel *et al*., 2006 [57]	English	RCT	Patients (≥16 years) with BPD	IG: 26 CG: 26	IG: Aripiprazole 15 mg/day CG: Placebo	8 weeks	Depression; Anxiety; Anger expression; NSSI	Aripiprazole significantly reduced anxiety and depression;	92.30%	Level 2
								Aripiprazole significantly improved core BPD symptoms (affect, impulse, aggression);		
								A numerical trend favoring reduced NSSI was observed, but without statistical confirmation due to measurement limitations		
Nickel *et al*., 2007 [58]	English	Prospective and observational follow-up study	Patients (≥16 years) with BPD	Initial sample: 52 Final analysis: 42 at first, 39 at second/third follow-up	Aripiprazole 15 mg/day	Naturalistic observation	Depression; Anxiety; Anger expression; NSSI	Aripiprazole-treated patients maintained significant improvements on all outcome scales over 18 months, with changes significantly greater than the ex-placebo group;	92.30%	Level 3
								A numerical reduction in self-injury was observed in the aripiprazole group compared to the control group (15.4% vs 42.3%), but this difference was not statistically tested		
Izumi *et al*., 2022 [59]	English	Case-control study	Patients with suicide attempts, deliberate self-harm, and controls with accidental injury/intoxication	Suicide attempts: 39 Deliberate self-harm: 29 Controls: 166	No intervention	Not applicable	Associations between serum/plasma levels of lithium and the outcomes of suicide attempt or deliberate self-harm	Higher lithium levels were associated with fewer suicide attempts and deliberate self-harm	94.87%	Level 4
Kanehisa *et al*., 2017 [60]	English	Case-control study	Lithium therapy–naive patients transferred to an emergency department due to intoxication or injury	Suicide attempt: 31 Self-harm: 21 Control: 147	No intervention	Not applicable	Association between serum lithium levels and suicide attempts or deliberate self-harm	Serum lithium levels were significantly lower in the suicide attempt group than in the control group (especially among males);	94.87%	Level 4
								No significant association was found between lithium levels and self-harm		
Cipriani *et al*., 2013 [61]	English	Systematic review and meta-analysis	Patients with mood disorders	6674 participants	Long-term lithium therapy	Treatment duration was long-term (minimum 3 months, mean follow-up 19 months)	Number of completed suicides; Episodes of deliberate self-harm; All-cause mortality	Lithium was more effective than placebo in reducing suicides and all-cause mortality;	97.43%	Level 1
								No clear benefit of lithium over placebo in preventing deliberate self-harm;		
								Lithium was more effective than carbamazepine in reducing deliberate self-harm		
Cullen *et al*., 2018 [62]	English	Open-label pilot study	Female adolescents and young adults (aged 13–21)	35 females enrolled, 24 completed the 8-week trial	NAC: 600 mg twice daily (weeks 1–2), 1200 mg twice daily (weeks 3–4), 1800 mg twice daily (weeks 5–8)	8 weeks	Frequency of NSSI episodes (per 2 weeks)	Significant reduction in NSSI frequency at weeks 6 and 8 compared to baseline; NAC was generally well tolerated	92.30%	Level 4
Serafini *et al*., 2018 [63]	English	Systematic Review	Patients with MDD, TRD, NSSI behavior, and/or suicidal behavior	560 participants	Buprenorphine: as monotherapy or combined with opioid antagonists (samidorphan, naloxone, or naltrexone)	Treatment duration across studies ranged from a single dose or 4–7 days to 12 weeks (3 months)	Depressive symptom severity; Suicidal ideation; Frequency of NSSI episodes	Buprenorphine, alone or combined with opioid antagonists, may significantly and sometimes rapidly reduce depressive symptoms, NSSI, and suicidal ideation in patients with TRD and other conditions	87.20%	Level 2

MDD, major depressive disorder; MADRS, Montgomery-Åsberg Depression Rating Scale; GAD-7, Generalized Anxiety Disorder 7-item Scale; NAC, N-acetylcysteine; TRD, treatment-resistant depression.

**Table 3.** **A summary of neuromodulation technology studies in this narrative synthesis**.

Author	Language	Study design	Study subject	Sample	Intervention	Intervention (+ follow-up)	Primary outcomes	Main findings	Percentage total score	Level
Zhao *et al*., 2023 [64]	English	Prospective intervention study + cross-sectional comparison	Adolescents with MDD and NSSI	IG: 21 CG: 31	IG: SSRIs + rTMS CG: SSRIs	8 weeks	EEG microstate parameters under emotional stimuli; HAMD; NSSI	MDD + NSSI adolescents showed abnormal EEG microstates (MS 3, 4, 6) under negative emotional stimuli;	92.30%	Level 3
								SSRIs + rTMS led to greater reduction in both depressive symptoms and NSSI behaviors compared to medication alone, accompanied by normalization of the abnormal microstate parameters		
Shen *et al*., 2025 [65]	Chinese	RCT	Female adolescents with bipolar depression and NSSI	IG: 15 CG1: 15 CG2: 15	IG: rTMS + Bipolar depression triple therapy	2 weeks	Anxiety; Depression; Suicide risk	rTMS + triple therapy significantly reduced anxiety/depression and suicide risk, with some effect on NSSI	92.30%	Level 2
					CG1: Bipolar depression triple therapy					
					CG2: Sham rTMS + Bipolar depression triple therapy					
Liu *et al*., 2024 [66]	English	Retrospective cohort study	Patients with MDD and NSSI	IG: 65	IG: Sertraline + rTMS	4 weeks	Cognitive function;	Sertraline + rTMS can significantly improve cognitive function, increase neurotransmitter and neurotrophic factor levels, and reduce inflammatory factors	82.05%	Level 3
				CG: 65	CG: Sertraline		Serum inflammatory factor;			
							Neurotransmitters;			
							Neurocytokines			
Lei *et al*., 2025 [67]	English	RCT	Young adults (aged 18–29) with a history of NSSI	IG: 15 CG: 15	IG: Single 20-minutes active tDCS CG: Single 20-minutes sham tDCS	Single-session intervention + Assessments were conducted at baseline, immediately post-intervention, and at 24 hours, 1 week, and 2 weeks post-intervention	Pain sensitivity; Rumination; NSSI	Active tDCS did not produce significant between-group differences in pain sensitivity compared to sham stimulation;	94.87%	Level 2
								Active tDCS led to a statistically significant reduction in rumination at both 1-week and 2-week follow-ups;		
								No significant effects were found on NSSI urges, NSSI resistance, self-efficacy in resisting NSSI, or self-criticism		
Tong *et al*., 2025 [68]	English	Single-blind RCT	Adolescents with NSSI	IG: 32 CG: 26	IG: Active α-tACS CG: Sham α-tACS	1 week + Assessments were conducted at Baseline, 1 day post-intervention, and 7 days post-intervention	Frequency of NSSI; Depression; Anxiety	Active α-tACS significantly reduced the frequency of NSSI, depression and anxiety compared to sham stimulation	87.20%	Level 3
Gorodetsky *et al*., 2025 [69]	English	Phase I, non-randomized pilot study	children (ages 7–14) with ASD and severe, treatment-refractory self-injurious behavior	IG: 6	IG: DBS (NAc)	12 months follow-up	Frequency of self-injurious behavior; Severity of self-injurious behavior	DBS markedly reduced self-injurious behavior frequency and severity and improved functioning	97.43%	Level 4
Baizabal-Carvallo *et al*., 2022 [70]	English	Cross-sectional study	Patients with TS	201 TS patients	Not applicable	Not applicable	Frequency of self-injurious behavior in TS	Among the small subset of patients who underwent DBS (n = 10), self-injurious behavior outcomes were: 2 had complete resolution, 7 had partial improvement, and 1 had no benefit	79.49%	Level 4
Keshtkar *et al*., 2011 [71]	English	RCT	Adult patients with MDD	IG: 40 CG: 33	IG: ECT CG: rTMS	ECT: 3 weeks rTMS: 10 days	Depression severity; Suicidal behavior	Both treatments significantly improved depression and suicidal behavior;	82.05%	Level 3
								ECT was more effective than rTMS in reducing depression scores and suicidal behavior in short term		
Salagre *et al*., 2022 [72]	English	Population-based observational study	Patients diagnosed with unipolar depression, bipolar disorder, psychotic disorder, or personality disorder	ECT group: 8843 (unipolar depression), 2713 (bipolar), 2692 (psychotic), 2085 (personality) Comparison groups: Multiple matched groups	ECT	Periods before and after ECT initiation (1 month, 3 months, 6 months, 1 year, 2 years)	Number of incidents of self-harm/suicide attempts	Substantial and rapid reduction in self-harm/suicide attempts after ECT initiation;	82.05%	Level 3
								In the first month post-ECT, events decreased by 72% to 83% across all diagnostic groups;		
								Reductions were sustained but attenuated over longer periods (up to 2 years)		
Rootes-Murdy *et al*., 2019 [73]	English	Retrospective study	Adolescents and young adults (aged 14–25) with treatment-resistant unipolar or bipolar depression	48 patients	IG: ECT	Mean ECT sessions: 12.6 (Overall), 15.4 (NSSI group), 11.1 (Non-NSSI group)	Treatment response; Remission; Switch to bilateral ECT; Length of inpatient stay; Number of ECT treatments	In the overall sample, NSSI was associated with more ECT treatments, longer hospital stays, and higher rates of switching to bilateral ECT;	79.49%	Level 4
								In female patients, NSSI was significantly associated with substantially lower odds of treatment response and remission, and required more treatments compared to females without NSSI		

rTMS, repetitive transcranial magnetic stimulation; EEG, electroencephalogram; tDCS, transcranial direct current stimulation; tACS, transcranial alternating current stimulation; ASD, autism spectrum disorder; TS, Tourette’s syndrome; ECT, electroconvulsive therapy; NAc, nucleus accumbens; HAMD, Hamilton Depression Rating Scale.

**Table 4.** **A summary of digital health technology-related studies in this narrative synthesis**.

Author	Language	Study design	Study subject	Sample	Intervention	Intervention (+ follow-up)	Primary outcomes	Main findings	Percentage total score	Level
Stallard *et al*., 2018 [29]	English	Case series study	Young people (aged 12–17)	40 participants	Usual CAMHS care + BlueIce	Assessments at baseline, 2 weeks (post-familiarization), and 12 weeks (post-use)	Frequency of self-harm; Depression; Anxiety; Acceptability and safety of the app	73% of recent self-harmers reported reduced self-harm;	87.20%	Level 4
								Significant reductions in depression and anxiety scores at 12 weeks;		
								High acceptability (93% used it, 88% wanted to keep it)		
Stallard *et al*., 2024 [74]	English	Mixed methods study (Content analysis of post-use qualitative interviews conducted within an RCT framework)	Adolescents (aged 12–17) with repeated self-harm	60 participants	Usual CAMHS care + BlueIce	A semi-structured telephone interview was conducted at 12 weeks	Acceptability; Use patterns; Safety	95% used BlueIce; 82% used it when thinking about self-harm; 77% reported it prevented ≥1 self-harm episode; No user reported BlueIce triggered self-harm	97.43%	Level 3
Burke *et al*., 2021 [75]	English	Prospective observational cohort study	University students (aged 18–26) with a history of repetitive NSSI	60 participants	Not applicable	Baseline assessment (emotional stop-signal task) followed by a 10-day EMA protocol with 3 random prompts per day	Intensity of NSSI urges	Higher momentary negative affect and urgency were independently associated with stronger NSSI urges;	79.49%	Level 4
								Deficits in emotional response inhibition to self-harm stimuli moderated the link between momentary negative affect and NSSI urge intensity		
Herzog *et al*., 2022 [76]	English	Prospective observational study	Adults with BPD	82 participants	Not applicable	7-day EMA	Change in suicidal ideation following episodes of NSSI	NSSI episodes were preceded by increases in suicidal ideation;	79.49%	Level 4
								NSSI episodes were followed by significant reductions in suicidal ideation in the subsequent hours		
Su *et al*., 2023 [77]	English	Longitudinal Cohort Study	Adolescents (age 14–17)	2809 participants	Not applicable	Not applicable	Self-harm and suicide attempt (past 12 months)	Random forest model outperformed models based solely on prior self-injury/suicide history	82.05%	Level 4
Lee *et al*., 2025 [78]	English	RCT	Adults (aged 19–50) with MDD	IG1: 19 IG2: 19 CG: 19	IG1: VR-based CBT IG2: TAU CG: Healthy controls	6 weeks + 4 weeks follow-up	Depressive symptoms; Suicidality	Depressive symptoms improved significantly over time in VR group and TAU group, with no significant difference between groups;	94.87%	Level 3
								The suicidality score decreased significantly only in VR group at follow-up		

CAMHS, child and adolescent mental health services; EMA, ecological momentary assessment; VR-based CBT, virtual reality-based cognitive behavior therapy.

## 3. Psychotherapy

To date, research specifically focused on treating NSSI has remained limited, although evidence supporting the effectiveness of psychotherapeutic interventions continues to grow [21, 79]. A recent systematic review underscored the distinct value of psychotherapy in addressing NSSI [21]. In clinical practice, the Association of the Scientific Medical Societies in Germany has recommended several core components for treating adolescents with NSSI [80]. These include establishing agreements on the management of suicidal behavior and NSSI, enhancing treatment motivation, providing psychoeducation, identifying factors that trigger or maintain self-injury, teaching alternative coping or problem-solving strategies, and addressing comorbid psychiatric disorders in accordance with relevant guidelines.

Among commonly used psychotherapies such as DBT, CBT, MBT, and ACT, these interventions generally aim to improve emotion regulation and reduce maladaptive coping strategies. Despite these shared elements, substantial differences remain in their efficacy and in the durability of their benefits [79]. Psychotherapy studies included in this review are presented in Table 1.

### 3.1 Dialectical Behavior Therapy

DBT, developed by American psychologist Marsha Linehan, is a cognitive-behavioral approach grounded in biosocial theory and dialectical philosophy. Initially designed for borderline personality disorder and recurrent suicidal behavior [81], DBT helps patients enhance emotion regulation, build interpersonal effectiveness, and improve social functioning across staged treatment modules. DBT has been widely adopted in later-phase interventions for emotion dysregulation and extreme behaviors, including suicidality and NSSI, particularly among adolescents [82].

Studies have indicated that DBT significantly reduces NSSI, reduces suicidal ideation, and enhances emotional regulation, particularly among adolescents with severe emotional regulation difficulties [33, 34, 36]. Berk *et al*. [34] reported that after DBT treatment, 74% of adolescents experienced a reduction in NSSI, and 63% experienced a reduction in suicidal ideation. It is important to note that DBT was significantly more effective than individual and group supportive therapy (IGST) in reducing NSSI (82.6% vs. 65.5%, *p* = 0.01) [83]. DBT has also demonstrated long-term advantages in reducing NSSI. In a randomized controlled trial, DBT for adolescents (DBT-A) led to a sustained reduction in NSSI frequency over a 3-year follow-up compared to treatment as usual (TAU) [37]. For adult patients, DBT has also demonstrated significant efficacy in improving NSSI behaviors, with no discernible difference in treatment outcomes between relatively short-term and long-term interventions [35].

Notably, not all DBT-informed interventions showed consistent benefits. DBT may demonstrate weaker or inconsistent efficacy in adolescents who exhibit prominent externalizing symptoms such as impulsivity and aggression, high rates of self-harm, or severe suicidal ideation [83]. Another randomized controlled trial found that brief DBT skills training for adults with recurrent suicidal ideation was associated with a higher risk of NSSI than was standard care [38]. This suggests that low-intensity, skills-only DBT formats may be insufficient, and potentially risky, for high-risk individuals, highlighting the need for careful adaptation and comprehensive treatment delivery.

### 3.2 Cognitive Behavioral Therapy

CBT is a structured, goal-oriented form of psychotherapy that aims to modify maladaptive thoughts and behaviors through cognitive restructuring and behavioral techniques, thereby alleviating emotional distress and related symptoms [84]. Its time-limited nature, focus on skill-building, practical applicability, and emphasis on relapse prevention have contributed to its widespread adoption across diverse patient populations and clinical conditions [84].

Studies have indicated that short-term, structured CBT can effectively reduce NSSI and associated emotional symptoms [39, 40]. Sinyor *et al*. [39] conducted CBT for a young group. Their findings indicated that brief cognitive behavioral therapy (BCBT) produced a significantly lower likelihood of repetitive NSSI during the acute phase than did minimally directive supportive psychotherapy (OR = 0.34, *p* < 0.05), with no suicide attempts reported throughout the intervention period [39]. Similarly, Lou *et al*. [40] conducted a 5-week intensive CBT (ICBT) program among Chinese adolescents with NSSI and observed significant improvements in positive emotion, NSSI function, and reductions in negative emotion, depression, anxiety, and self-injurious behaviors compared to TAU (all *p* < 0.05).

However, a recently published meta-analysis suggested that CBT may demonstrate comparatively lower efficacy relative to other psychotherapeutic interventions for NSSI, and in some cases even trended toward poorer outcomes [21]. This discrepancy may be attributed to structural differences in treatment approaches. Traditional cognitive restructuring in CBT requires repeated reflection and practice, which may not swiftly resolve the intense emotional reactions experienced by individuals engaging in NSSI [85].

The Cutting-Down Program (CDP), a structured intervention that integrates elements of CBT and DBT, has been developed specifically for adolescents with repetitive NSSI [86]. Kaess *et al*. [41] reported that comparable to treatment-as-usual, NSSI frequency was reduced (70.3% vs. 73%), alongside reductions in suicidal attempts and depressive symptoms, although between-group differences were not significant. Notably, CDP achieved these outcomes in fewer sessions, highlighting its potential as a time-efficient intervention [41]. A subsequent follow-up study by Rockstroh *et al*. [42] spanning 2–4 years demonstrated sustained benefits; NSSI frequency was further reduced by 84%. Moreover, each additional outpatient session during follow-up was associated with a 5.5% reduction in NSSI risk, underscoring the value of maintenance support [42].

### 3.3 Mentalization-Based Treatment

Mentalization-based treatment (MBT) is a psychodynamically oriented intervention rooted in developmental psychopathology, attachment theory, and the concept of mentalization—the capacity to interpret one’s own and others’ mental states. It aims to enhance self and emotion regulation, showing particular utility in addressing emotional dysregulation, impulsivity, and interpersonal dysfunction, especially in borderline personality disorder [87].

Evidence regarding the efficacy of MBT in NSSI remains mixed. In a randomized controlled trial by Griffiths *et al*. [43], MBT-Adolescent integrated (MBT-Ai) showed reductions in self-harm and related emergency visits that were comparable to TAU reductions, with no significant intergroup differences. Mentalizing ability was further identified as a significant predictor of self-harm and hospitalization, underscoring its relevance in NSSI behavior [43]. However, two other studies reported limited efficacy for MBT in reducing NSSI, attributing the results to factors such as treatment format, adolescent-specific characteristics, and outcome measurement issues [45, 46].

A recent randomized controlled trial comparing MBT-A and the Unified Protocol for Adolescents (UP-A) found that both interventions significantly reduced NSSI [44]. However, UP-A achieved greater improvements in emotional dysregulation [44]. Although the MBT and UP groups showed similar short-term effects, over follow-up (6 to 36 months) the MBT group exhibited a gradual rebound in NSSI behaviors, whereas the UP group showed a slower and less pronounced recurrence, suggesting that the long-term maintenance effects of MBT may be relatively limited [47].

### 3.4 Acceptance and Commitment Therapy

ACT is a mindfulness-based, contextual, behavioral approach designed to enhance psychological flexibility by promoting present-moment awareness, acceptance of difficult emotions, cognitive defusion, values clarification, and committed action. For individuals with NSSI, who often exhibit cognitive rigidity and resort to extreme behaviors to alleviate emotional distress, ACT provides an alternative to experiential avoidance and supports more adaptive responses to negative internal experiences [88, 89].

Studies have shown that ACT can enhance positive emotions and psychological flexibility in adolescents, reduce the frequency and intensity of stressful life events, alleviate depressive and anxiety symptoms, and significantly reduce the severity and incidence of NSSI (12.5% vs. 32.5%, *p* < 0.05) [24, 48].

These findings were corroborated by an additional randomized controlled trials, which indicated that ACT facilitates acceptance of negative emotions and thoughts, strengthens present-focused awareness and self-awareness, improves psychological flexibility, thereby contributing to a reduction in NSSI behaviors [49].

## 4. Pharmacotherapy

Current clinical guidelines emphasize that pharmacotherapy for NSSI should be administered as an adjunct to psychotherapy rather than as a stand-alone intervention [80]. This approach is particularly critical in adolescent patients, though short-term medication may be considered during acute crises, such as periods of severe tension or intense urges to self-harm [80]. Recent evidence has suggested that medications with potential efficacy in NSSI include selective serotonin reuptake inhibitors (SSRIs), serotonin-norepinephrine reuptake inhibitors (SNRIs), and atypical antipsychotics. Pharmacotherapy studies included in this review are presented in Table 2.

### 4.1 Antidepressants

Several antidepressants have been evaluated for their potential role in reducing NSSI, often in combination with psychotherapy. Liu *et al*. [50] found that sertraline combined with DBT achieved higher NSSI cessation than sertraline treatment with CBT at 6-month follow-up (57.8% vs. 32.6%, *p* < 0.05), along with greater reductions in anxiety and depression. Other antidepressants (e.g., fluvoxamine and escitalopram) combined with CBT have also been reported to alleviate the severity of depression and anxiety in depressed adolescents, reduce NSSI, enhance psychological resilience, and yield better overall treatment outcomes [52, 53].

A meta-analysis indicated that fluoxetine treatment combined with CBT was superior to fluoxetine alone in reducing depressive symptoms, NSSI or suicidal behavior, adverse events, and one-year relapse [51]. However, Davey *et al*. [27] reported contradictory results, noting that fluoxetine treatment plus CBT did not improve depressive symptoms, suicidal ideation, or suicidal behaviors more than fluoxetine treatment plus placebo therapy, and was associated with increased incidence of NSSI, particularly in participants under 18 years.

### 4.2 Atypical Antipsychotics

Atypical antipsychotics have been investigated as potential therapeutic options for NSSI, often as adjuncts to antidepressant treatment. A randomized controlled trials demonstrated that treatment with the combination of quetiapine and sertraline is more effective than sertraline monotherapy in reducing NSSI frequency, improving emotion regulation, and alleviating co-occurring anxiety and depressive symptoms in adolescent populations, with a favorable safety profile [54].

Low-dose olanzapine (2.5–5 mg) has also shown effectiveness in managing NSSI. Clinical trials indicated that olanzapine augmentation of sertraline contributed to reduced self-injurious and impulsive behaviors, with a relatively rapid onset and minimal adverse effects [55, 56]. In a double-blind, placebo-controlled study, aripiprazole treatment over 8 weeks was associated with a reduction in self-harm incidents, from 7 at baseline to 2 during treatment [57]. An 18-month follow-up of the same cohort further showed that the aripiprazole group maintained significantly fewer self-harm episodes than did the placebo group (15.4% vs. 42.3%), without an increased risk of suicidal behavior [58].

### 4.3 Mood Stabilizers

The potential role of mood stabilizers, particularly lithium, in the management of NSSI has been explored in observational studies, although the findings remain inconsistent. One investigation found that higher levels of naturally occurring lithium were significantly associated with reduced risk of both suicide attempts and NSSI. After adjusting for sex and age, log-transformed serum lithium concentrations were negatively correlated with NSSI across all multivariate models (OR <1), supporting a potential protective role of lithium against self-injurious behaviors [59].

In contrast, another study reported no statistically significant difference in log-transformed serum lithium levels between the NSSI group and controls (*p* = 0.057). Even after adjusting for sex and age, the odds ratio for lithium levels in the NSSI group was 1.91 (*p* = 0.492), suggesting that there was no significant protective effect against NSSI [60]. This conclusion aligned with a previous meta-analysis [61] that indicated limited efficacy of lithium in preventing NSSI.

### 4.4 Other Medications

Several other pharmacological agents have been explored for their potential in managing NSSI. In an open-label pilot study, treatment with N-acetylcysteine (NAC) led to a significant reduction in the frequency of NSSI among adolescents and young adults, alongside reductions in depressive and general psychopathological symptoms, though not in impulsivity. NAC was well-tolerated, with no serious adverse events reported [62].

Studies have suggested that low-dose buprenorphine and low-dose naltrexone may reduce the frequency and severity of NSSI, with good tolerability and an overall acceptable safety profile [63, 90].

## 5. Neuromodulation Technology

Emerging evidence has suggested that the pathophysiology of NSSI involves dysregulation across several core neurophysiological domains, including emotion processing, reward feedback, pain perception, and impulse control, each associated with distinct abnormalities in brain regions and neural circuits [20]. To target these circuit-level dysfunctions, a range of neuromodulation techniques has been explored. These include non-invasive or minimally invasive approaches such as transcranial magnetic stimulation, transcranial electrical stimulation, deep brain stimulation, and electroconvulsive therapy, which aim to normalize neural activity in circuits implicated in NSSI. Neuromodulation technology studies included in this review are presented in Table 3.

### 5.1 Transcranial Magnetic Stimulation

Transcranial magnetic stimulation (TMS) is a non-invasive neuromodulation technique that uses pulsed magnetic fields to induce electrical currents in targeted cortical regions, thereby modulating neuronal excitability, promoting neuroplasticity, and influencing neurotransmitter systems such as dopamine [91]. Repetitive TMS (rTMS), the most widely used clinical form, applies repeated pulses at varying frequencies to either enhance or suppress neural activity in specific brain areas [92].

Several clinical studies on patients with NSSI have evaluated rTMS, primarily applied over the dorsolateral prefrontal cortex (dlPFC). A study in adolescents with depression and NSSI found that rTMS combined with pharmacotherapy significantly reduced depressive symptoms and NSSI behaviors compared with medication alone (*p* < 0.05) [64]. A similar finding was reported in adolescents with bipolar depression and NSSI, in which active rTMS combined with triple pharmacotherapy produced the greatest short-term improvements in anxiety, depression, and suicide risk (*p* < 0.05), indirectly contributing to a lower incidence of NSSI [65]. In addition, rTMS combined with sertraline significantly improved cognitive function, increased neurotransmitter and neurotrophic factor levels, and reduced cytokine levels. This research indirectly suggested that rTMS combined with sertraline treatment offers broad therapeutic benefits for patients with depression accompanied by NSSI [66].

### 5.2 Transcranial Electrical Stimulation

Transcranial electrical stimulation (TES) encompasses a group of non-invasive brain modulation techniques, with transcranial direct current stimulation (tDCS) and transcranial alternating current stimulation (tACS) being the most widely studied. tDCS modulates cortical excitability by applying a weak direct current to alter neuronal resting membrane potentials [93]. Brunoni *et al*. [94] reported that both tDCS alone and combined with sertraline treatment effectively regulated prefrontal cortex function in depressed patients. It reduced cognitive impairments (e.g., attention deficits) and emotional processing (e.g., pessimistic thoughts and suicidal ideation), with combined treatment yielding superior relief in core depressive symptoms [94].

Wang *et al*. [95] reported that dlPFC-targeted tDCS combined with quetiapine in bipolar depression produced an early and sustained anti-suicidal effect with a favorable safety profile, but did not confer additional antidepressant benefit. Conversely, a separate randomized trial found no significant improvement in depressive symptoms or suicidal ideation with tDCS across session durations [96]. In a study by Lei *et al*. [67], a single active tDCS session was associated with significantly less rumination in individuals with NSSI than was sham stimulation, although it did not affect impulsivity, resistance to self-injury, self-efficacy, or pain sensitivity.

In contrast, tACS modulates brain activity by entraining neural oscillations through the application of alternating current at specific frequencies [97]. Tong *et al*. [68] demonstrated that α-tACS applied over the dlPFC alleviated attentional bias toward NSSI-related cues and reduced self-injurious behaviors in adolescents. Moreover, the degree of attentional bias improvement at one day post-intervention effectively predicted the reduction in NSSI after seven days of consecutive α-tACS sessions [68].

### 5.3 Deep Brain Stimulation

Deep brain stimulation (DBS) is an invasive neuromodulation technique that involves the surgical implantation of electrodes to deliver controlled electrical pulses to specific brain nuclei or circuits. In the field of psychiatry, DBS remains in early-stage clinical exploration, with preliminary evidence supporting its potential for conditions such as obsessive-compulsive disorder and treatment-resistant depression [98].

Direct clinical studies evaluating DBS specifically for NSSI are few. However, evidence from patients with comorbid self-injurious behaviors suggested that DBS may reduce self-harm as it addresses the primary disorder. For instance, a study of 201 patients with Tourette’s syndrome (TS), 16.9% of whom exhibited self-injurious behavior, found that of those who received DBS (10 patients), 2 achieved complete remission of self-injury, 7 showed partial improvement, and only 1 did not benefit [70].

In a non-randomized phase I pilot study, 6 children with autism spectrum disorder and severe NSSI received DBS targeting the nucleus accumbens. After 12 months of follow-up, significant reductions were observed in the frequency, severity, and repetitiveness of NSSI (by 32.1%, 38.2%, and 27.7%, respectively). The behavior’s functional role also showed an improving trend, and the treatment was well-tolerated, with no serious adverse events and a marked improvement in quality of life [69].

### 5.4 Electroconvulsive Therapy

Electroconvulsive therapy (ECT) is a well-established psychiatric treatment that involves the application of electrical currents to one or both cerebral hemispheres to induce generalized seizures and muscle relaxation under general anesthesia [99]. ECT demonstrates significant effectiveness in mitigating suicide risk [100, 101] and is widely used in patients with suicidal behavior. One randomized controlled trial indicated that ECT yielded superior short-term antidepressant effects and greater reduction in suicidal behavior than did repetitive transcranial magnetic stimulation (rTMS) [71].

However, evidence regarding the efficacy of ECT specifically for NSSI remains limited and somewhat inconsistent. One study reported that ECT significantly reduced the frequency of self-harm and suicide attempts across multiple psychiatric diagnoses [72]. In contrast, a retrospective analysis found that female patients with a history of NSSI had significantly lower rates of treatment response and remission after right unilateral ECT than did those without such a history [73]. Those findings suggested that although ECT may help alleviate self-injurious behaviors in certain clinical contexts, its role in the management of NSSI requires further systematic investigation.

## 6. Digital Health Technology

The stigma surrounding self-injury often deters individuals with NSSI from seeking traditional professional help, due to fears of judgment, privacy concerns, or being perceived as attention-seeking [102]. One study indicated that one-third to one-half of adolescents with NSSI do not seek help for these behaviors, and many instead turn to online platforms for expression and support [102].

Mobile health (mHealth) offers a promising alternative to conventional face-to-face interventions, providing high accessibility, adaptability, and the potential to optimize user engagement and adherence [103, 104]. Available mHealth modalities include smartphone applications, supportive text messaging, wearable sensors for tracking physiological symptoms, and virtual therapy sessions [105]. Digital health technology-related studies included in this review are presented in Table 4.

### 6.1 Mobile Applications

Stallard and colleagues [29], along with young people who have a history of self-harm, co-developed a smartphone application named “BlueIce” for managing NSSI in adolescents. In a 12-week study involving 40 adolescents aged 12–17, significant reductions in self-harm behaviors and reductions in depression and anxiety symptoms were observed. The application was well-accepted and demonstrated good safety according to feedback from both users and clinicians [29].

In a subsequent mixed-methods study focusing on BlueIce for self-harm prevention, Stallard *et al*. [74] reported that 82% of adolescents with NSSI used the application when considering self-harm. Notably, 77% indicated that BlueIce helped prevent at least one self-harm episode [74]. However, 70% of participants reported either continuing to self-harm or not using the app during self-harm incidents. Commonly cited reasons included an inability to focus on the app during acute emotional distress, a negative mindset, and premeditated self-injury [74]. Those findings suggested that although digital self-help tools such as BlueIce offer valuable support, they may not fully substitute for in-person crisis intervention.

### 6.2 Remote Monitoring

Remote monitoring technologies enable real-time tracking of emotional states and risk factors in naturalistic settings, offering new avenues for intervention. Hetrick *et al*. [106] co-developed a mood monitoring and management application with clinicians and young adults (aged 18–25) experiencing depression, suicidal ideation, or self-harm. The application features onboarding, mood tracking, distraction techniques, brief interventions, and a safety alert function [106]. This approach not only improves communication between clinicians and young people regarding symptoms and treatment progress but also creates opportunities for timely, evidence-based interventions.

Ecological momentary assessment (EMA), a real-time data collection method, captures individuals’ immediate experiences in daily life, thereby minimizing recall bias [107]. Burke *et al*. [75] used EMA to monitor adolescents with a history of repetitive NSSI and found that momentary negative affect and urgency independently and positively predicted the intensity of NSSI urges.

In a 7-day EMA study of patients with borderline personality disorder, Herzog *et al*. [76] observed that each 1-point increase in suicidal ideation from pre-episode to the moment of NSSI was associated with a 15.2% increase in the likelihood of self-injury. Additionally, suicidal ideation decreased by an average of 1.77 points after NSSI episodes, suggesting a short-term alleviating effect of self-injury on such ideation [76].

### 6.3 Intelligent Assistance

AI has demonstrated significant potential in identifying early warning signs of self-harm and suicide by analyzing large-scale datasets to generate personalized risk predictions and tailored intervention strategies [108]. In a study by Su *et al*. [77], a random forest machine-learning model was developed to predict self-harm and suicide attempts in adolescents. The model achieved area under the curve (AUC) values of 0.740 for self-harm and 0.722 for suicide attempts, outperforming prediction approaches based solely on historical data [77].

In another application, Lee *et al*. [78] conducted a randomized controlled trial in patients with major depressive disorder (MDD). Results indicated that virtual-reality-integrated cognitive behavioral therapy (VR-based CBT) was not inferior to pharmacotherapy in alleviating depressive and anxiety symptoms and reducing perceived stress, and also significantly lowering suicide risk, with no serious adverse events reported [78]. Although research on VR-based interventions specifically for NSSI remains limited, these findings suggest a promising direction for future studies to explore its efficacy in self-injury prevention and treatment.

## 7. Discussion

This narrative review synthesized current evidence on interventions for NSSI, spanning psychological, pharmacological, neuromodulatory, and digital health approaches. However, the evidence exhibited significant heterogeneity in terms of outcome measures, age ranges, comorbidities, and follow-up durations. Based on this, we have developed a clinically oriented summary table designed to enhance the clinical readability and practicality of the results (Table 5, Ref. [21, 24, 33, 34, 35, 36, 37, 39, 40, 41, 42, 43, 44, 45, 46, 47, 48, 49, 53, 55, 64, 65, 67, 68, 69, 74, 75, 76, 77]).

**Table 5.** **Overview of interventions for NSSI**.

Intervention	Short-term effects	Longer-term effects	Age groups with strongest evidence
DBT/DBT-A	Robust reduction in NSSI frequency and self-harm episodes in adolescents and adults with emotion dysregulation and BPD traits [33, 34, 36, 37]	Maintenance of reduced NSSI over 1–3 years in adolescent samples; non-inferiority of 6- vs 12-month DBT in adults [35, 37]	Adolescents and adults with BPD traits or severe dysregulation
CBT/BCBT/ICBT/CDP	Short-term decrease in NSSI and self-harm; faster early response but sometimes smaller effect sizes than DBT [39, 40, 41]	Limited data; CDP follow-up suggests sustained reductions but convergence with TAU [41, 42]	Mainly adolescents and young adults
ACT/UP-A	Short-term improvements in emotion regulation and NSSI frequency when delivered as brief adjuncts to usual care [24, 48, 49]	Some attenuation of gains over 6–12 months; long-term relapse risk remains [44]	Predominantly adolescents and young adults
MBT/MBT-A/MBT-G	Mixed results; some reduction in self-harm, but often comparable to TAU [43, 45, 46]	Long-term follow-up indicates partial relapse of gains in both adolescent and adult samples [44, 47]	Adolescents with BPD traits; adults with BPD + ASPD
SSRIs/SNRIs	In network meta-analyses, possible short-term increase in NSSI/self-harm in youth; improvement in depressive symptoms [21]	Reduction in NSSI/self-harm after >3 months in some samples [21, 53]	Mainly adolescents and young adults; some adult data
Atypical antipsychotics (e.g., quetiapine, low-dose olanzapine)	Short-term improvements in NSSI frequency and comorbid mood/impulsivity [55]	Little long-term data; potential metabolic and extrapyramidal adverse effects	Adolescents and young adults with affective lability and impulsive aggression
Mood stabilizers, N-acetylcysteine, opioid receptor modulators	Preliminary improvements in self-harm or suicidal behavior in small adult samples	Long-term safety and durability unknown	Adults with treatment-resistant mood disorders and chronic self-harm
rTMS	Short-term reduction in depressive symptoms and, in some studies, NSSI or self-harm when combined with antidepressants [64, 65]	Insufficient long-term data	Adults and older adolescents with mood disorders
tDCS/tACS	Modest effects on rumination and attentional bias; mixed or absent effects on NSSI urge itself [67, 68]	Minimal evidence beyond a few weeks	Older adolescents and young adults
DBS	Marked reduction in NSSI frequency and severity in very small adult pilot samples [69]	Preliminary 12-month data suggest sustained benefit in some cases	Adults with severe, treatment-resistant NSSI and comorbid OCD-like symptoms
ECT	Unknown	Unknown	Unknown
Mobile apps (e.g., BlueIce)	Good acceptability and perceived usefulness; reduction in some self-injury episodes, especially as self-management tools [74]	Long-term engagement and impact remain unclear	Adolescents
EMA and digital phenotyping	Clarifies temporal dynamics of negative affect, urges, and NSSI episodes; improves risk detection [75, 76]	Prognostic value over 1–2 years demonstrated in some cohorts [77]	Adolescents and young adults

OCD, obsessive-compulsive disorder.

### 7.1 Psychotherapy

Among psychological interventions, DBT demonstrated the most consistent benefits, particularly for adolescents with severe emotion dysregulation and recurrent NSSI [37, 82, 83]. From a neurodevelopmental perspective, heightened emotional vulnerability in adolescents may reflect earlier maturation of the limbic system relative to prefrontal regulatory regions, which may make them more likely to adopt NSSI as a functional coping strategy, particularly in invalidating environments [81]. DBT addresses this mechanism through structured training, helping individuals balance acceptance and change to reduce reliance on self-injury [81]. DBT not only effectively improves NSSI in the short term but also offers long-term therapeutic benefits for individuals, whether adolescents or adults. Notably, however, DBT shows limited efficacy in youths with prominent externalizing symptoms, such as impulsivity or aggression, or those with intense suicidal ideation. In some cases, low-intensity DBT may even increase NSSI risk [38, 83], underscoring the importance of accounting for treatment-response heterogeneity.

Although some studies supported the short-term efficacy of CBT in reducing NSSI [39, 40], a recent meta-analysis suggested that CBT may be significantly less effective than DBT and could even elevate NSSI risk relative to placebo [21]. NSSI behaviors may occur during peaks of negative emotions. The emotional-cascade model proposes that rumination and negative affect amplify each other [109]. When this cycle of rumination and negative affect intensifies, individuals may seek more effective ways to distract themselves from these overwhelming emotions; NSSI is one such method [110]. CBT requires repeated practice and reflection to alter cognitive and behavioral patterns. Consequently, during emotional peaks in individuals with NSSI, CBT’s delayed effects cannot promptly regulate their emotions, leading them to abandon treatment or persistently rely on self-harm behaviors. In contrast, DBT’s timely coping strategies enable individuals with NSSI to adjust during emotional peaks, thereby reducing the occurrence of NSSI.

MBT shows some capacity to reduce self-harm, but long-term outcomes have been suboptimal, and there is evidence of symptom rebound over time [47]. First, this may have been related to the inclusion of samples in which differences in enrollment criteria, disease severity, and comorbid diagnoses could have led to inconsistent efficacy of MBT. Second, it may have been related to the format of MBT treatment; group-based therapy may not be able to monitor individual emotional changes in real time. Furthermore, the core of MBT lies in enhancing mentalization abilities. Although this may yield short-term improvements, achieving more stable behavioral change requires extended training periods. ACT, which targets psychological flexibility, offers another valuable alternative, particularly in mitigating NSSI severity through reduced experiential avoidance [88]. Overall, skills-based protocols such as DBT remain the first-line psychological intervention for NSSI, combining immediate coping tools with a structured therapeutic framework.

### 7.2 Pharmacotherapy

In the current treatment landscape, pharmacotherapy serves primarily as an adjunctive approach. Clinically, when NSSI is primarily driven by emotional or psychiatric symptoms, treating the underlying mental disorder may yield greater benefits than addressing the NSSI itself. Antidepressant monotherapy appears to have limited efficacy for core NSSI symptoms, but it may exert synergistic effects when combined with psychotherapy [50, 52, 53, 111]. This benefit appears mediated through alleviation of comorbid depressive and anxiety symptoms, thereby indirectly reducing self-injury frequency, especially in cases of adolescent depression accompanied by NSSI [54, 55, 56].

However, pharmacological interventions require careful risk-benefit analysis. Antidepressants carry a documented risk of increased suicidal ideation and NSSI, particularly in pediatric populations [26]. That concern prompted the U.S. Food and Drug Administration to issue a black box warning in 2004 for antidepressant use in children and young adults [112]. Consistent with this, one clinical trial reported not only the absence of improvement in suicidal thoughts and behaviors but also actually twice as many incidences of NSSI in participants under 18 as in older patients [27]. That finding has practical implications for clinicians. Clinicians should prioritize the combination of antidepressants and psychotherapy, rather than monotherapy or avoidance of antidepressants; for children and adolescents, close monitoring and a safety management plan should be implemented during treatment [113]. Augmentation with low-to-moderate doses of atypical antipsychotics may further enhance therapeutic response, improving both affective symptoms and reducing NSSI behaviors [54, 55, 56].

The evidence for mood stabilizers remains nuanced. Lithium has been shown to have efficacy in suicide prevention [59], yet its utility for NSSI remains controversial, possibly reflecting differing motivational underpinnings between suicidal behavior and NSSI [61]. One study indicated that suicide is closely associated with impulsivity and aggression [114]. Lithium may reduce suicide risk by decreasing impulsive aggressive behavior and stabilizing mood through mechanisms including serotonin regulation, enhancement of glutamatergic and GABAergic system function, and effects on neuroplasticity [115]. NSSI is typically regarded as an emotion-regulation strategy, the functional tendency of which is to maintain behavior through immediate negative reinforcement, exhibiting high heterogeneity in terms of motivation, intent, and comorbid disorders [116]. NSSI episodes are often triggered by brief emotional surges or interpersonal triggers [110], rather than necessarily stemming from impulsive aggressive states. Therefore, lithium salts have relatively limited direct effects on NSSI. In clinical treatment, the use of lithium salts should prioritize suicide prevention as the primary goal, with NSSI management serving as an adjunctive objective.

Emerging agents such as NAC [62] and opioid-receptor modulators [63, 90] show preliminary promise, suggesting novel neurobiological pathways for intervention. Nevertheless, their clinical translation requires further validation through rigorously designed randomized controlled trials.

### 7.3 Neuromodulation Technology

Neuromodulation techniques represent an emerging frontier in NSSI treatment by directly targeting aberrant neural circuits implicated in its pathophysiology. The dlPFC, a key hub for emotion regulation and cognitive control, is frequently dysregulated in individuals with NSSI [117, 118]. rTMS applied to the dlPFC has demonstrated promise in modulating these networks and reducing self-injury [64, 65, 66]. Current rTMS protocols, however, largely rely on broad anatomical targeting. Precision targeting of specific neural circuits is emerging as a research hotspot. Combined with magnetic resonance imaging, navigation-guided rTMS enables precise stimulation of designated brain regions. Emerging approaches combining accelerated high-dose regimens with fMRI-guided neuronavigation, such as functional connectivity-targeted intermittent theta-burst stimulation, have achieved remission rates exceeding 90% in treatment-resistant depression within five days [119, 120]. This paradigm of personalized, circuit-informed neuromodulation holds significant potential for translation to NSSI populations. Therefore, future research should combine high-dose stimulation with fMRI-guided targeting to deliver individualized rTMS treatment to specific brain regions and neural circuits in patients with NSSI.

Among TES techniques, tDCS modulates cortical excitability to influence cognitive and affective processes [94, 95]. In depression, tDCS combined with pharmacotherapy has demonstrated significant therapeutic effects [94]. tACS can entrain neural oscillations to reduce NSSI-related attentional bias [68]. TES offers advantages such as non-invasiveness, minimal side effects, and excellent safety and tolerability. However, it also has limitations that include prolonged treatment duration, relatively short-lasting therapeutic effects, and significant interindividual variability. Clinical application should emphasize strict adherence to treatment parameters and vigilant monitoring of side effects, particularly in pediatric patients [121].

DBS offers a potential therapeutic approach for severe self-injurious behavior. DBS indirectly modulates the amygdala-hippocampal core node and engages the emotional-impulse control neural circuit involving the anterior cingulate cortex and medial orbitofrontal cortex. This improves patients’ emotional responses, reduces impulsive aggressive behavior [122]. DBS has been shown to have encouraging results in reducing self-injury frequency and severity [69]. However, the available evidence largely comes from patients with neurodevelopmental disorders and is mainly derived from case reports or single-arm pilot studies, with a lack of randomized controlled trials and limited long-term data on safety and efficacy. Therefore, as an invasive modality, DBS necessitates careful weighing of surgical risks and ethical considerations, particularly in children and adolescents.

ECT has been widely used in patients with depression who exhibited suicidal behavior due to its ability to reduce suicide risk rapidly, alleviate depressive symptoms [71]. However, evidence supporting the efficacy of ECT in patients with NSSI remains insufficient. One study indicated that individuals with a history of NSSI exhibit poorer response to ECT [73]. Therefore, although ECT can rapidly reduce suicide risk, its use in NSSI should still be approached with caution and further targeted research is needed. Currently, non-invasive neuromodulation, particularly optimized rTMS, should be regarded as an emerging physical intervention for severe or treatment-resistant NSSI, with preliminary evidence suggesting a favorable balance between efficacy, safety, and the potential for greater precision, although its scalability and routine use remain to be established.

### 7.4 Digital Health Technology

Digital health technologies offer a promising complement to conventional NSSI interventions through accessibility, anonymity, and real-time support, features that align closely with the habits and preferences of digitally engaged youth. Evidence has suggested that well-designed mobile apps can effectively reduce emotional symptoms and self-harm behaviors among adolescents [29]. Although the application offers some benefits in addressing self-harm behaviors, it also has certain limitations. First, its utility is limited during acute self-harm crises, as emotional intensity and cognitive overload often impede engagement [74]. Second, most people are unaware of or not using available mental health apps or self-help tools [123]. Third, most mobile applications lack rigorous evaluation, and their content often fails to align with relevant evidence-based guidelines [124, 125]. Fourth, mobile applications still pose privacy and data security risks [104]. Therefore, a comprehensive privacy and security policy should be established before promoting the application.

EMA enables real-time mood tracking and dynamic risk prediction, offering valuable insights into proximal triggers of NSSI [75, 76]. EMA research has found that NSSI provides short-term relief from suicidal ideation, supporting the functional model of NSSI as negative reinforcement. That serves as a reminder that screening for suicide risk should include an assessment of NSSI and prompt intervention to prevent patients from relying on NSSI to manage suicidal states [76]. Meanwhile, artificial intelligence supports early identification through multivariate predictive modeling [77]. However, one recent review suggested that these approaches may be limited by issues related to data quality, bias, poor generalizability, and ethical and privacy concerns, which may restrict their direct application in clinical settings [126]. Virtual reality creates immersive environments for exposure-based therapy and safety planning, with demonstrated reductions in suicide risk [78]. However, direct evidence for virtual reality–based interventions in NSSI remains limited. The clinical generalizability, cost-effectiveness, and acceptability among adolescents experiencing acute emotional distress require further validation [127]. Collectively, digital innovations appear promising for supporting more personalized and scalable approaches to NSSI care, although the evidence base is still preliminary.

### 7.5 Synthesis

**Fig. 1.**
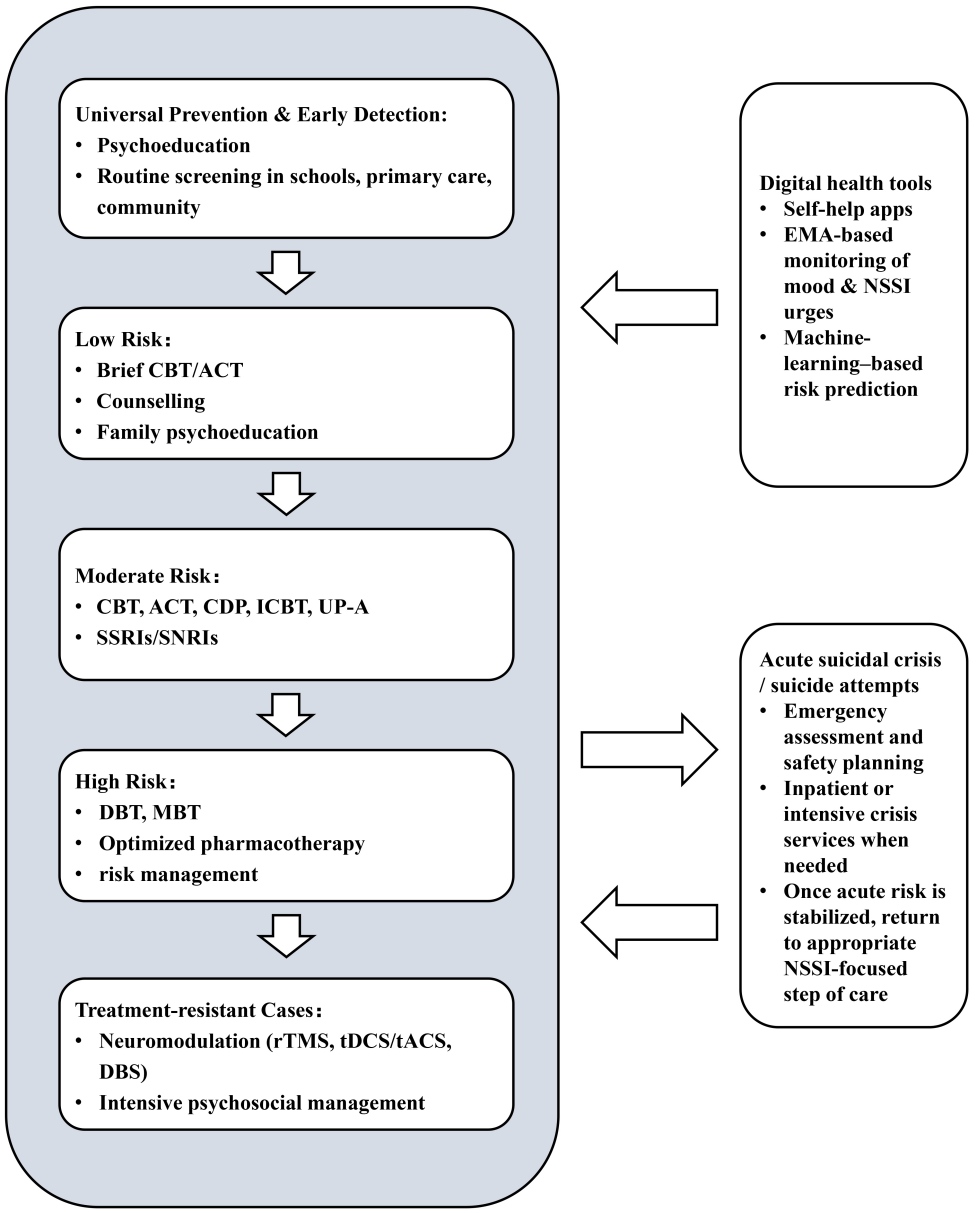
**Multimodal care model for NSSI**. This diagram illustrates a stepwise treatment framework for NSSI, progressing from universal prevention and early screening to pathways for treatment-resistant cases, with corresponding interventions displayed at each tier. Digital health tools serve as auxiliary modules across all tiers, supporting assessment, monitoring, self-management, and relapse prevention—functioning as enhancements rather than substitutes for traditional therapies. Neuromodulation technologies are primarily employed at higher tiers as adjunctive interventions for treatment-resistant NSSI or mixed suicidal cases, requiring careful application in specialized or research settings. When suicidal behavior occurs, crisis management such as emergency care or hospitalization is necessary. Following stabilization of acute risk, treatment focused on NSSI resumes at the appropriate tier.

By synthesizing evidence on interventions for NSSI, we observed encouraging outcomes across various intervention strategies. Based on the reviewed evidence, we constructed a stepwise, personalized, and holistic tiered framework for NSSI interventions, providing guidance for clinicians, educators, and policymakers (Fig. 1). It is important to note that in cases of suicide attempt or acute suicidal crisis, corresponding crisis management procedures must be initiated immediately. NSSI-focused interventions should only be reintroduced once the situation has stabilized.

## 8. Limitations

Several limitations of this review should be acknowledged. First, as a narrative review, this study lacked a systematic retrieval strategy, which may have introduced selection bias. Despite efforts to include high-quality and recent literature, the sheer volume and distribution of studies may have resulted in the omission of some important findings. Considerable heterogeneity was observed across included studies in terms of intervention protocols, sample characteristics, and outcome measures, limiting direct comparability of findings and precluding quantitative meta-analysis. Although we applied a pragmatic quality appraisal using percentage quality scores and levels of evidence, we did not perform a formal, tool-based risk-of-bias assessment for each individual study. Therefore, our conclusions should be interpreted with caution in light of potential biases in the underlying literature. Second, some studies included in this review primarily focused on broad self-harm or suicidal behaviors as their primary outcomes, which, to some extent, limited their direct comparability with NSSI. Third, the exclusion of publications in languages other than English or Chinese may have introduced language bias. Fourth, evidence for several emerging interventions, including deep brain stimulation, novel pharmacological agents, and virtual reality, remains preliminary and is often based on small pilot studies with limited follow-up, limiting conclusions regarding long-term efficacy, safety, and sustainability.

## 9. Future Research Directions

Despite the emergence of several promising interventions, several research priorities warrant emphasis. First, head-to-head comparative trials with sufficient statistical power should be conducted in clearly defined NSSI samples, rather than being confined to broad self-injury samples, to more clearly delineate the relative efficacy and cost-effectiveness of different treatments across adolescent and adult cohorts. Second, future studies should identify moderators and mediators of treatment response, including age, sex, comorbidities, and functional types of NSSI, to enable more precise matching of interventions to patient subgroups. Third, neurostimulation and pharmacotherapy trials should extend follow-up durations, adopt standardized NSSI and suicide behavior outcomes, and enhance safety monitoring, particularly when adolescents are involved.

## 10. Conclusions

In summary, a multimodal and individualized approach is recommended for the management of non-suicidal self-injury. Psychotherapy, particularly DBT, currently has the strongest empirical support among available psychosocial interventions, with growing evidence indicating its efficacy in improving emotion regulation, distress tolerance, and reducing self-injurious behaviors. Recent evidence has suggested that pharmacotherapy is best used as an adjunct for addressing comorbid mood symptoms or acute crises, and should be prescribed with caution, particularly in adolescents, given the potential increase in NSSI risk associated with some antidepressants and the need for careful risk–benefit evaluation. Emerging agents such as N-acetylcysteine and opioid receptor modulators show preliminary promise, yet require further validation of their efficacy and safety.

Neuromodulation techniques represent an emerging circuit-based intervention strategy, with preliminary evidence suggesting potential benefits for some individuals with NSSI. Future efforts should focus on optimizing stimulation parameters, developing individualized targeting protocols, and advancing non-invasive technologies to enhance treatment precision. Digital health tools offer significant potential in improving accessibility, enabling real-time monitoring, and providing personalized support, especially for younger populations. However, they should be viewed as complementary to, rather than replacements for, traditional approaches, particularly in crisis situations where professional intervention remains essential.

Ultimately, an integrated framework for NSSI intervention should be established, centering on psychotherapy while strategically incorporating pharmacotherapy, neuromodulation, and digital tools to achieve precise, effective, and sustainable outcomes.
